# New Perspectives on Aspirin and the Endogenous Control of Acute Inflammatory Resolution

**DOI:** 10.1100/tsw.2006.192

**Published:** 2006-08-31

**Authors:** Thea Morris, Melanie Stables, Derek W. Gilroy

**Affiliations:** Centre for Clinical Pharmacology and Therapeutics, Division of Medicine, 5 University Street, University College London, London WC1E 6JJ, UK

**Keywords:** inflammation, resolution, nitric oxide, NSAIDs

## Abstract

Aspirin is unique among the nonsteroidal anti-inflammatory drugs in that it has both anti-inflammatory as well as cardio-protective properties. The cardio-protective properties arise form its judicious inhibition of platelet-derived thromboxane A_2_ over prostacyclin, while its anti-inflammatory effects of aspirin stem from its well-established inhibition of prostaglandin (PG) synthesis within inflamed tissues. Thus aspirin and the other NSAIDs have popularised the notion of inhibiting PG biosynthesis as a common anti-inflammatory strategy based on the erroneous premise that all eicosanoids are generally detrimental to inflammation. However, our fascination with aspirin has shown a more affable side to lipid mediators based on our increasing interest in the endogenous control of acute inflammation and in factors that mediate its resolution. Epi-lipoxins (epi-LXs), for instance, are produced from aspirins acetylation of inducible cyclooxygenase 2 (COX-2) and together with Resolvins represent an increasingly important family of immuno-regulatory and potentially cardio-protective lipid mediators. Aspirin is beginning to teach us what nature knew all along — that not all lipid mediators are bad. It seems that while some eicosanoids are pathogenic in a variety of diseases, others are unarguable protective. In this review we will re-count aspirins colorful history, discuss its traditional mode of action and the controversies associated therewith, as well as highlight some of the new pathways in inflammation and the cardiovascular systems that aspirin has recently revealed.

## INTRODUCTION

How and why does a drug like aspirin, originating from its willow bark, myrtle and poplar tree derived ancestor salicylate, whose natural analgesic, anti-thrombotic, anti-pyretic, anti-inflammatory and anti-eodema properties have been known for years, still draw so much attention? What else can this compound divulge? Surprisingly, a convincing mechanism of action of aspirin is still lacking. Over the past few decades the scientific community has avidly been trying to uncover how and in which capacity this drug actually works. Such knowledge is particularly important in view of the accumulation of evidence suggesting that inhibition of PG synthesis by aspirin, put forward convincingly by Vane[1], is not the sole way in which aspirin exerts its anti-inflammatory effects. The history and theories exposed over the years regarding aspirin and its modes of action are reviewed with an emphasis on its capacity as a potent anti-inflammatory drug. It seems likely that its mechanism of action will be disease specific and interestingly may have as much to do with triggering anti-inflammatory/pro-resolving signals as it does with inhibiting pathways that initiated the inflammatory response in the first instance.

## ASPIRIN'S HUMBLE BEGINNINGS

Extracts of willow bark, meadow sweet and wintergreen were used in antiquity to alleviate the suffering caused by rheumatic pain and the discomfort associated with childbirth as well as the use of poplar tree extracts for the treatment of eye inflammation. Not surprisingly, the common constituent of all these medicinal plants was found to be *salicin*, later renamed salicylate[2]. Years later, in a letter to the Royal Society in London in 1763, the Reverend Edmund Stone reported how he successfully treated 50 fellow parishioners suffering from rheumatic fever with a crude extract of willow bark, an effect confirmed over 100 years later by T.J. MacLagan who reported how salicylate lowered body temperature while reducing pain and swelling caused by rheumatism[3]. This clearly demonstrated the anti-pyretic, analgesic and anti-inflammatory properties of salicylate. Both believed in the "*doctrine of signatures*" which claims that the cure for an ailment can be found in the areas where that disease occurs most frequently, which in the case of Stone and MacLagan was the abundance of Willow trees in dampened regions, which were believed to cause rheumatic diseases. Heartened by Carl Gerhardt's chemical analysis of salicylate, Hermann Kolbe managed to identify the basic structure of salicylate as an *o*-hydroxybenzoic acid and together with Lautemann in 1859 successfully synthesised salicylate[2]. By the time MacLagan's work was published in 1876[3], other physicians were using the new synthetic aspirin. Indeed, it was in this year that the first clinical trial in Berlin, Germany, demonstrated salicylate's anti-rheumatic effects. 15 years later von Heyden, Kolbe's student, established the synthetic process by which salicylate could be made on an industrial scale leading to the widespread availability of salicylate in its pure form. Successful and effective though salicylate was, it was not without its undesirable side effects[2] The acetylation of salicylate in 1897 by Felix Hoffman working for Bayer, Germany resulted in the birth of aspirin, an equally efficacious drug with comparatively fewer side effects than salicylate (Figure 1) The following 60 years saw extensive usage of aspirin but will little clue as to how it works.

## ASPIRIN: 100 YEARS LATER

Nowadays the importance of aspirin lies in its established anti-inflammatory and cardio-protective properties as well as its emerging role in protecting against cancer and neurodegenerative diseases. However, its true mode of action in all these settings remains unclear. Throughout the later half of the 20$^{th}$ century aspirin research was a melting pot of hypotheses and speculations as to the mechanisms by which aspirin exerted its anti-inflammatory and protective effects. Suggested was its capacity to uncouple oxidative phosphorylation[4], inhibit dehydrogenase enzymes dependent on pyridine nucleotides[5] and inhibit some amino-transferases and/or decarboxylases[6]. One of the most convincing theories in the 1960s was aspirin's ability to inhibit proteases located at inflammatory sites and known to cause tissue damage associated with chronic inflammatory diseases. However, the concentration of aspirin required to bring about these *in vitro* effects was considerably higher than the plasma level of aspirin reached after ingesting anti-inflammatory doses of the drug. Moreover, inhibiting any or all of these candidate pathways could not explain aspirin's ability to alleviate the heat, redness, swelling and pain classically defined by Celsus as the cardinal signs of inflammation[7]. Thus, more than 50 years following the birth of aspirin no convincing mechanism as to how it regulates the inflammatory response had been found

1

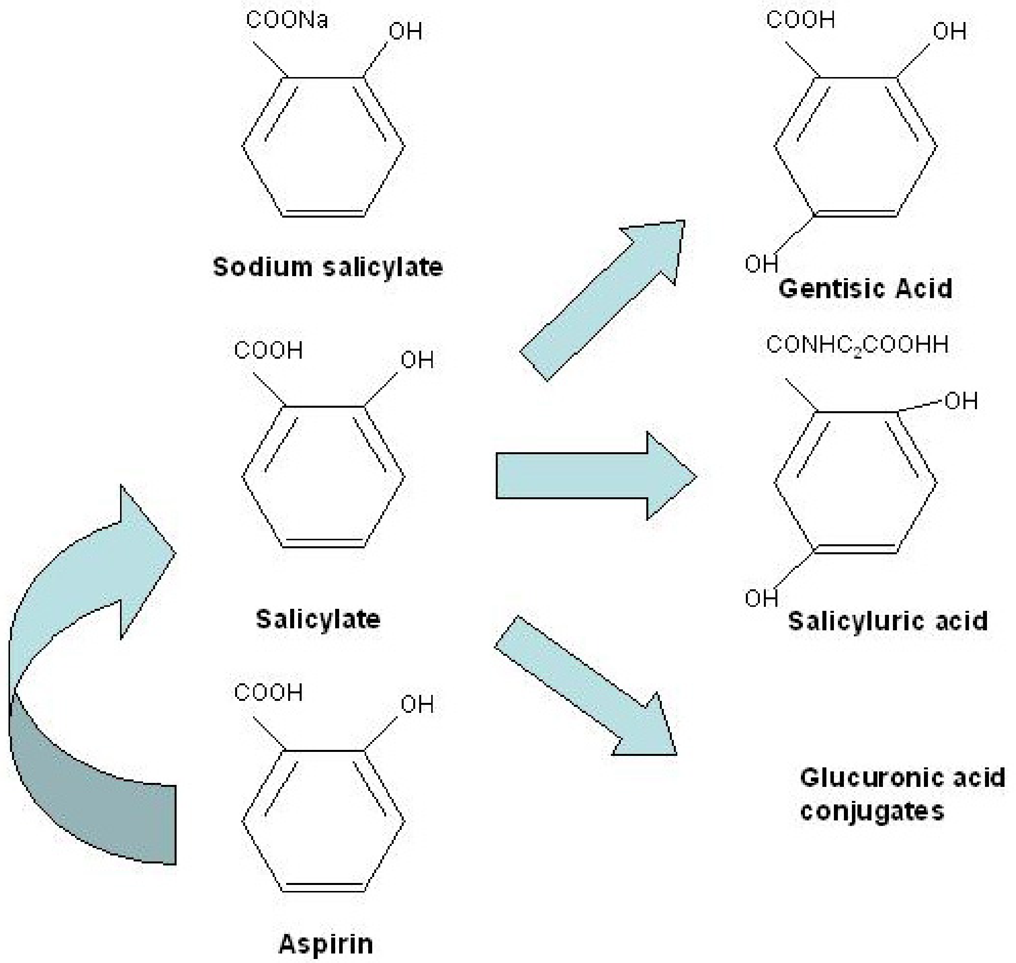

**FIGURE 1.** *In vivo* pathway of aspirin metabolism. After oral administration, aspirin is rapidly absorbed and first pass metabolism induces the formation of salicylates with a short half-life in the order of 15 to 20 mins, detectable before salicylates are measureable. Clearance of salicylate and therefore aspirin, can occur via 5 pathways, with all metabolites being excreted via the kidney.

until the work of Vane revealed that aspirin and other aspirin-like drugs, including indomethacin and salicylate, inhibited tissue PG synthesis[8]. The emerging face of the PGs was one of mediating pain, swelling and indeed the whole gambit of inflammatory symptoms immortalised by Celsus[7]. Concomitantly, Smith and Willis showed similar inhibitory effects of aspirin on prostacyclin synthesis by platelets[9] while Flower and Vane tested various NSAIDs showing that at therapeutic concentrations PGs were inhibited and that this inhibition correlated with reduction in inflammation[10]. Certainly, PGs are released in response to inflammatory stimuli and enhance the cardinal signs of inflammation. For example, $PGE_2$ is a potent vasodilator produced in sufficient amounts to cause erythema associated with acute inflammation. It also causes increased vascular permeability leading to oedema formation in synergy with histamine or bradykinin collectively providing compelling evidence that the mechanism of action of aspirin lay in its ability to block the PG synthetic pathway. Thus it appeared that the enigma was solved.

This theory however bares inconsistencies due to PG's inability to regulate leukocyte recruitment, which is more the domain of the leukotrienes (LTs), in particular 5-lipoxygenase (LOX)-derived $LTB_4$. These are grounds enough to suggest that inhibition of PG biosynthesis is not sufficient to explain aspirin's ability to inhibit leukocyte accumulation during inflammation. Moreover, aspirin's unique cardio-protective properties (unlike other NSAIDs) raises another issue pertaining to its ability to inhibit platelet thromboxane synthesis (75mgs) at doses considerably lower than its typical anti-inflammatory effects (1g), thus aspirin can inhibit PGs synthesis at doses that are not anti-inflammatory in man suggesting that other pathways that control the inflammatory response may be altered by aspirin.

## MECHANISM BY WHICH ASPIRIN MAY WORK IN INFLAMMATION

### Some Early Ideas

Although most research concerning the anti-inflammatory effects of NSAIDs and particularly aspirin is based on their ability to inhibit COX-derived PG biosynthesis, it was during the 1990s that salicylate was shown to be a transcription-modulating drug, acting both dependently and independently of COX 2 inhibition. For instance, salicylate was shown to suppress NF-kappaB activation[12] and NF-kappaB mediated inflammatory responses such as chemokine and adhesion molecule gene expression[13,14] as well as iNOS expression[15]. On the other hand, salicylate having anti-inflammatory properties comparable to those of aspirin[16] and the ability to inhibit PG biosynthesis *in vivo*[17] was found to suppress COX 2 protein expression *in vitro* as well as in experimental animal models of acute inflammation[18]. These claims have been disputed by Mitchell and colleagues who suggest that direct COX-2 enzyme inhibition is responsible for the anti-inflammatory effect of salicylate in an *in vivo* model of LPS-dependent COX 2 induction in the rat[19]. These conflicting results show that the effects of salicylate are separate to aspirin and are most likely variable depending on experimental models and conditions, but still underline the importance of this compound in the inhibition of PG biosynthesis and its anti-inflammatory capacity.

Although largely accepted since Vane's demonstration that aspirin was directly involved in inhibiting PG synthesis[11], some groups suggested that not all anti-inflammatory effects could be accounted for by the inhibition of either or both COX isoforms. One such theory arose from Weissmann's observation that therapeutic serum levels of salicylate, poor inhibitors of COX 1 and COX 2 enzyme activity in man, correlate better with clinical anti-inflammation than serum concentrations of aspirin[20]. Subsequent studies demonstrated that aspirin and sodium salicylate are anti-inflammatory in their capacity to interfere with neutrophil activation and ability to interact with endothelial cells at concentrations known to not inhibit PG synthesis[21]. Salicylates, but not indomethacin and piroxicam, inhibit oxidative phosphorylation, resulting in ATP hydrolysis and release of adenosine followed by a clear reduction in neutrophil aggregation. The importance of aspirin induced extracellular adenosine release during inflammation was confirmed by an *in vivo* model of carrageenan-induced inflammation in which neutrophil accumulation after aspirin treatment was reduced in mice deficient in COX 2 and NF-kappaB. Aspirin was therefore proposed to act *via* a pathway independent of the suppression of the PG biosynthesis[22]. One of the potential shortfalls of this work is that these studies were carried out using the air pouch model of acute leukocyte-mediated inflammation. This requires the formation of an air pouch capsule lining that orchestrates the inflammatory response subsequent to infection or injury. Using this model either in animals knocked out for a particular gene inherent to the inflammatory response or dosing animals with drugs known to alter the immune response during the formation of this capsule lining, which was the regime employed by Cronstein and colleagues, is potentially erroneous as it is not known what effect the absence of COX or presence aspirin would have on capsule formation and subsequent elicited inflammatory response.

Another route through which aspirin may act is in the induction of a sub-set of heat-inducible proteins called Heat Shock proteins (HSP), especially Hsp70. Heat shock proteins are protective chaperones regulated by heat or stressed activated heat shock transcription factors (HSF) that interact with a particular regulatory element, the heat shock element (HSE) in the promoter region of the HSP gene[23]. The activation mechanism of the HSF is poorly understood. However it is known that this process involves the hyperphosphorylation of the protein complex once trimerisation of the HSF monomers has occurred[24,25]. *In vitro* studies conducted during the early 1990s demonstrated the ability of certain

NSAIDs (aspirin and indomethacin) to lower the temperature threshold through which Hsp70 transcription occurs and potentiate its expression in part due to their ability to augment the level and duration of HSF-binding activity and hyperphosphorylation in response to heat stress and/or partially activate HSF1 to a DNA binding form, even in the absence of heat[26,27,28]. Further *in vivo* experiments were performed to gain a greater understanding into this novel mechanism of aspirin and perhaps more importantly elucidate a more concise pathway of action to show the direct physiological and clinical relevance aspirin has in treating a variety of human ailments. One such study, performed by Fawcett and colleagues in 1997[29] used mild hyperthermically challenged rats treated with aspirin reproducing the effects seen previously; an up-regulation of Hsp70 gene expression during heat stress. However, surprisingly it was unable to directly alter the DNA binding properties of HSF1, suggesting that the mechanism serving to produce this effect is different *in vivo*. Additional work, uncovered the ability of aspirin to enhance the core body temperature in these heat stressed rats, therefore providing an explanation for the up-regulation of Hsp70 in this setting.

## More Recent Perspectives

This overview of proposed mechanisms of action of aspirin paints a colourful picture of a drug with diverse actions and elusive modes of action. However, in recent times a more exciting aspect of its pharmacology stems from its ability to trigger the synthesis of lipid-derived mediators called aspirin-triggered lipoxins (ATL) specifically 15-epi-LX $A_4$ and $B_4$ which, along with their native counterparts $LXA_4$ and $LXB_4$, are switched on during the onset and resolution phases of inflammation. These anti-inflammatory lipid mediators of the eicosanoid family act via a G-protein coupled receptor termed ALX/FPRL-1 expressed on neutrophils, monocytes, epithelial cells and activated T cells[30] as well as enterocytes and fibroblasts[31]. LXs also signal through the aryl hydrocarbon receptor (AhR), which is a ligand activated transcription factor whose expression has been found to be upregulated subsequent to LX treatment. It is a receptor known to induce enzymes that accelerate the metabolism and clearance of planar lipophilic substances such as polycyclic hydrocarbons.

The LXs are formed through three distinct transcellular biosynthesis involving 5, -12 and -15-LOX as well as COX 2. The first pathway involves the insertion of $O_2$ to the OH group on carbon 15 of arachidonic acid by 15-LOX within eosinophils, monocytes or epithelial cells. Following its release from these cells and entry to either PMNs or monocytes, a 5,6-epoxytetraene is generated by 5-LOX, which is then hydrolysed within these recipient cells by either $LXA_4$ hydrolase or $LXB_4$ hydrolase to bioactive $LXA_4$ and $B_4$, respectively[32] (Figure 2A). The second route of LX biosynthesis results from the generation of $LTA_4$ by 5-LOX within leukocytes, its release and subsequent uptake by platelets and metabolism by 12-LOX to $LXA_4$ and $B_4$ [33,34] (Figure 2B).

Perhaps one of the most exciting aspects of the mechanism of action of aspirin stems from its ability to trigger LX synthesis (so-called aspirin-triggered epi-LXs) as a result of acetylating the active site of COX 2 in endothelial or epithelial cells, a property not shared with other NSAIDs. This results not in the inhibition of COX 2 enzyme activity, as might be expected, but in the conversion of arachidonic acid to (15 *R*) hydroxyeicosatetraenoic acid (15*R*-HETE), which is rapidly metabolised in a transcellular manner by leukocyte 5-LOX to 15-epi-LX $A_4$ or $B_4$[35] (Figure 2C).

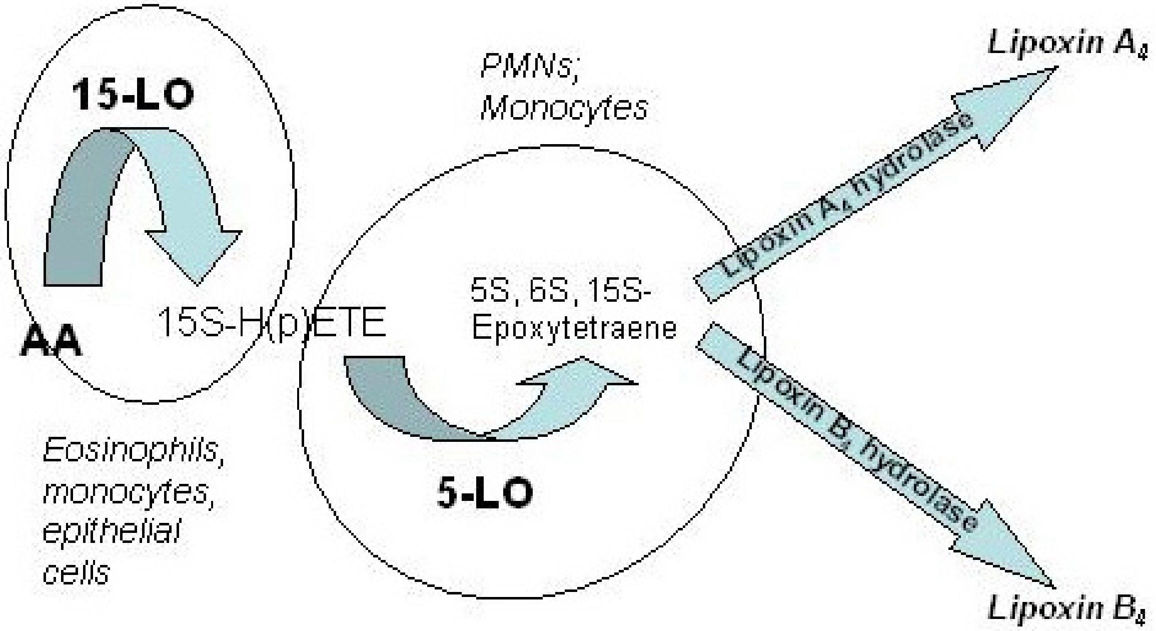

15-LO
AA
15S-H(p)ETE
Eosinophils, monocytes, epithelial cells
PMNs; Monocytes
5S, 6S, 15S-Epoxytetraene
5-LO
Lipoxin A4 hydrolase
Lipoxin B4 hydrolase
Lipoxin A4
Lipoxin B4

**2A**

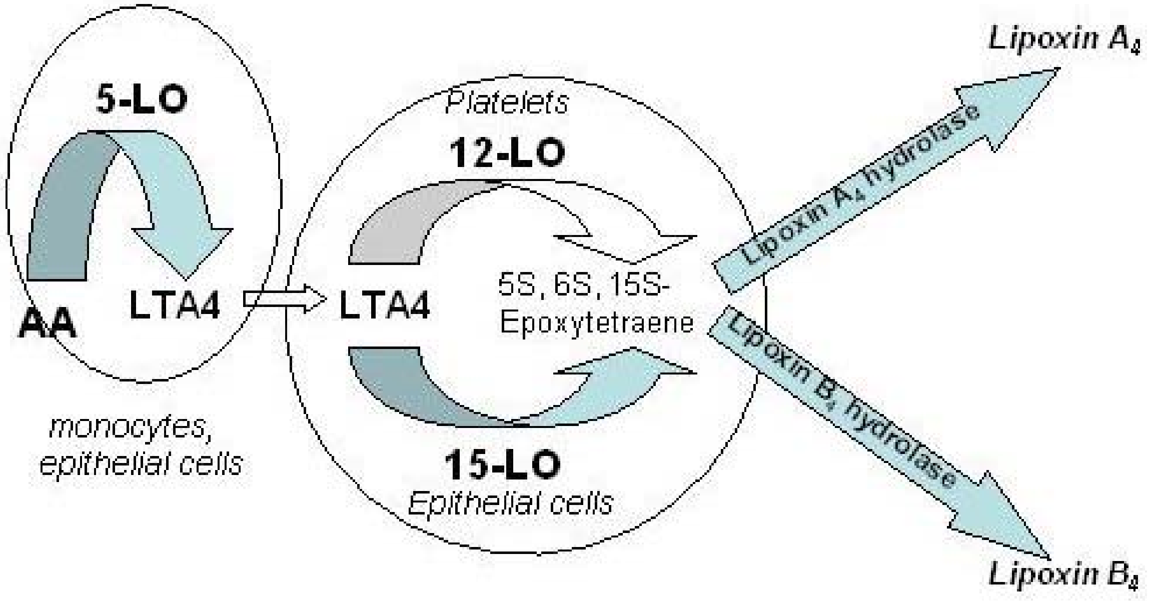

5-LO
AA
LTA4
monocytes, epithelial cells
Platelets
12-LO
LTA4
5S, 6S, 15S-Epoxytetraene
15-LO
Epithelial cells
Lipoxin A4 hydrolase
Lipoxin B4 hydrolase
Lipoxin A4
Lipoxin B4

**2B**

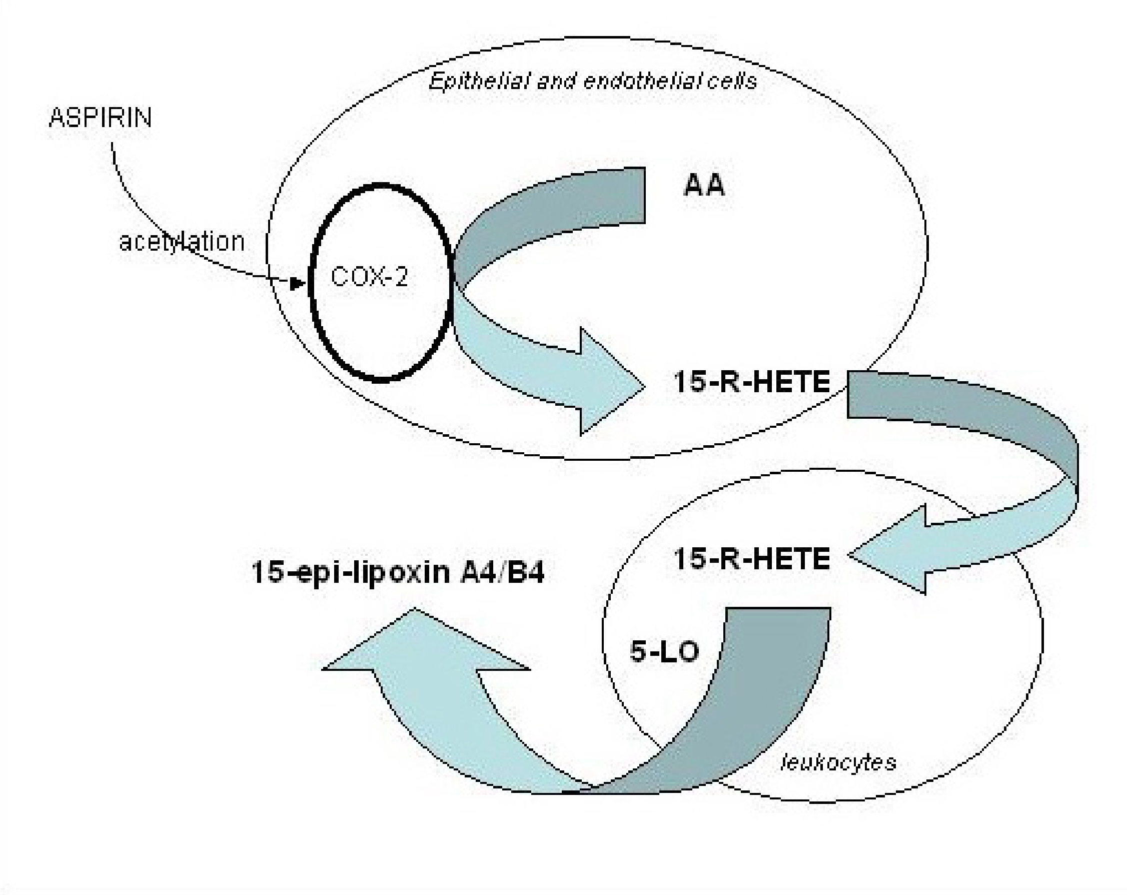

**2C**

**FIGURE 2.** Arachidonic acid metabolism by LOX and COX family of enzymes. (A) The first pathway consists of the insertion of molecular oxygen into carbon 15 of arachidonic acid via 15-lipoxygenase. The intermediate 15S-H(p)ETE serves as an immediate substrate for the 5-lipoxygenase present in PMNs and monocytes inducing the formation of epoxide intermediate 5S, 6S, 15S epoxytetraene, further metabolized by hydrolases (lipoxin A4 and B4 hydrolases) to forming native lipoxin $A_4$ and lipoxin $B_4$. (B) This second route consists in 5-lipoxygenase mediated synthesis of LTA4 in monocytes and epithelial cells. LTA4 is released and further metabolized by platelet 12-lipoxygenase or epithelial cell 15-lipoxygenase to the epoxide intermediate5S, 6S, 15S epoxytetraene further hydrolised by hydrolases $A_4$ and $B_4$. (C) The remaining arachidonic acid initiated pathway leading to lipoxin biosynthesis is dependent on aspirin-mediated acetylation of COX-2 which converts arachidonic acid to its 15R-HETE conformation, which when released from endothelial cells can be metabolized to 15-epi-lipoxin A4 and B4 by leukocyte 5-lipoxygenase.

The three pathways described above can operate independently or simultaneously within the vasculature. GM-CSF primed neutrophils, for example, recruited to the inflammatory site can interact with platelets[34]. After platelets adhere to the neutrophil surface, active PMNs generate $LTA_4$, which is released and transformed by platelet 12-LOX to generate LXs. Within the vasculature, the aspirin-triggered LX pathway can also be initiated when activated endothelial cells interact with adherent neutrophils to generate 15-epi-LXs. Leukocytes interacting with epithelial cell surfaces, as in the case of respiratory, renal or gastrointestinal inflammation, can also generate LXs through bi-directional routes in which (15S)-HETE and (15R)-HETE are released by epithelial cells and converted to LXs by neutrophils. The other component of this bi-directional interaction can involve neutrophil-released $LTA_4$, which is converted by 15-lipoxygnease in epithelial cells, in particular tracheal epithelial cells, to generate LXs.

Evidence placing LXs at the centre stage of inflammation research came from the detection of elevated levels of $LXA_4$ in *in vivo* inflammatory settings of experimental immune complex glomerulonephritis[36], in pleural exudates upon allergen challenge in rats[37], in ischemic lungs and hind limb ischemia reperfusion models[38], as well as during microbial infection with Toxoplasma gondii-exposed murine model[39,40]. Elevated levels of aspirin-induced 15-epi-$LXA_4$ were also detected in models of murine peritonitis[41], dorsal air pouch[42], in rat kidney[36] and liver[43]. In contrast, studies focusing on non-resolving inflammatory responses such as asthma[44] or cystic fibrosis[45,46], have shown that biosynthesis of $LXA_4$ is somehow compromised. This strengthens the hypothesis that LXs are essential regulators of inflammation and fibrotic events. Furthermore, recently Wu and colleagues gave a detailed account of the pathway whereby $LXA_4$ carries out its inhibitory effects on connective tissue growth factor (CTGF)-induced human lung fibroblast proliferation[47], making LXs primordial anti-fibrotic mediators.

LXs are anti-inflammatory in their ability to inhibit neutrophil chemotaxis and diapedesis through postcapillary venules and therefore entry into inflamed tissues in animal models (for review[48]). Patcha and colleagues showed that $LXA_4$ inhibits $LTB_4$-induced integrin clustering and neutrophil motility thereby preventing acute inflammatory events[49]. In contrast, $LXA_4$ also stimulates chemotaxis and adherence of monocytes to sites of inflammation, an essential mechanism in the processes of wound healing and clearance of an inflamed site. Attracted monocytes differentiate into macrophages capable of phagocytosing apoptotic neutrophils in a non-phlogistic manner[50], particularly during the resolution phase of inflammation where LXs promote monocyte chemotaxis and adherence without inducing neutrophil degranulation or release of other reactive oxygen species[51,52]. 15-epi-LXs formed subsequent to aspirin administration share these properties in addition to other downstream beneficial effects that include increased vasorelaxation[53], prostacyclin synthesis[54] and induction of nitric oxide synthesis by endothelial cells[55]. The benefits of LXs are virtually unquantifiable and need to be exploited. The synthesis of stable analogues is common practice and a necessary tool to continue the investigative process into their mechanisms of action. An essential step however, is the exploration of the aspirin-triggered-LX pathway in which intrinsic mechanisms still need to be unravelled.

The pathway triggered by aspirin acetylation of COX-2 and subsequent ATL synthesis has been well characterised in experimental models of acute inflammation[56], tissue injury[57] and in clinical settings[58,59]. Indeed aspirin has been shown to increase both urine and plasma levels of 15-epi-LX in healthy human volunteers and in rodents. An interesting study led by Chiang and colleagues showed that volunteers administered low dose aspirin (81mg/kg) once daily for 8 weeks, showed a significant increase in plasma 15-epi-$LXA_4$ levels and decreased thromboxane levels, an effect which was not reproduced at the higher doses of 325mg/kg and 650mg/kg of aspirin. This finding confirms that higher doses of aspirin do not potentiate aspirin induced cardio-protection, and suggests that the low dose aspirin-induced cardio-protective events typically afforded by 75-105mg/kg may be mediated by the local generation of ATL at the leukocyte/endothelial cell interface[59]. Could such low doses also be anti-inflammatory?

This work was followed up by an investigation as to whether aspirin's effects were gender dependent. Subjects were allocated into their gender (male vs female) and age groups. Analysis of aspirin-triggered 15-epi-$LXA_4$ levels showed a positive (increase of 0.37ng/ml per decade) and a negative (decrease of 0.29ng/ml per decade) correlation with age in females and males respectively. This clearly reflects that low dose aspirin has a gender specific impact on ATL formation which could account for the observed gender-dependent clinical benefits of aspirin[60].

Emphasis on the importance of ATLs in inflammation has been substantiated by experimental models showing that they actively dampen the host response and induce pro-resolution mechanisms. In a model of dermal inflammation, it was evidenced that topical application of LX stable analogues to mouse ears inhibited both PMN infiltration and vascular permeability changes. In addition, an ATL analogue administration to a TNF-alpha induced air-pouch model inhibited leukocyte recruitment and this effect was more potent than local delivery of aspirin[61]. A rat peritonitis model also showed a potent inhibitory effect of ATL analogue on neutrophil infiltration and protein extravasation when given intravenously[62]. This review of LX-induced anti-inflammatory effects requires one to delve into the signalling pathways via which such effects may occur.

## ATLs: Signalling Through the Lipoxin Receptor

Members of the LX family are known to signal through two receptors. The AhR nuclear receptor or the membrane bound receptor, coupled to a G protein known as ALX which was the first LOX-derived eicosanoid receptor to be isolated and cloned in human, mouse and rat tissues. Studies in transgenic models have shown that it is selective for $LXA_4$ (not for $LXB_4$, $LTB_4$, $LTD_4$ or $PGE_2$) with high affinity (Kd=1.7nM)[63]. Evidence that protective effects of LX and ATLs were ligand/receptor mediated arose from studies in a transgenic model of zymosan peritonitis in which neutrophil infiltration was decreased in transgenic mice over-expressing the human ALX[64]. These mice also showed increased sensitivity to suboptimal doses of ATL analogues emphasising the importance of this receptor in the downstream anti-inflammatory events triggered by LXs. Although this receptor is selective for $LXA_4$, 15-epi-$LXA_4$ and their analogues, other endogenous structurally distinct peptide ligands for ALX have been identified. These include annexin 1 and derived peptide Ac2-26, MHC binding peptide, anti-microbial peptides, HIV envelope proteins, neurotoxic peptides, acute phase proteins and bacterial peptides. Peptide ligands have been shown to stimulate chemotaxis and calcium mobilisation *in vitro*[65]. Although peptide and lipid ligands act via the same receptor, affinities and downstream effects differ greatly, rendering ALX an extremely versatile receptor central to the control of inflammatory responses and the outcome of ligand binding is likely to differ depending on the nature of the ligand. Interestingly however, the glucocorticoid-derived Annexin 1 and its derived peptides, Ac2-26 inhibit neutrophil diapedesis and have in common with ATLs their ability to limit neutrophil infiltration, reduce pro-inflammatory mediator production in a model of murine dorsal air pouch, enhance detachment of adherent leukocytes in the mesenteric circulation[66] and promote phagocytosis of apoptotic neutrophils by human macrophages[67], all central to resolution of inflammation. The findings relating ATL and ALX signalling with their ability to induce resolution raised the question as to what signalling pathway could be involved.

The second type of receptor that LXs are capable of binding to is the ligand-activated transcription factor, the aryl hydrocarbon receptor (AhR), which is a member of the basic helix-loop-helix superfamily of DNA binding proteins that in its inactive state resides in the cytosol complexed to a heat shock dimer. When bound by a ligand, it undergoes a transformation allowing it to dissociate from the complex and enter the nucleus to form a heterodimer with the AhR nuclear translocator (ARNT). This heterodimer is then capable of binding to specific sequences on the DNA, initiating the activation of a collection of genes primarily involved in the xenobiotic metabolism. Despite much research into the putative role of the AhR within the cell, there is still some controversy surrounding it. For simplicity the most understood effect lies in the ability of it to trigger the induction of enzymes that can accelerate the metabolism and clearance of planar lipophillic substances that have been ‘dubbed’ as being members of a large class of environmental carcinogens. There has also been evidence to suggest that the AhR plays an important part in regulating normal growth and differentiation, parallel to the removal of carcinogens. Using AhR null mice and cultured AhR-deficient Hepatoma cells laboratories were able to present data showing their mice to have an increased foetal mortality rate and depressed immune systems as well as evidence to suggest a partial block of the G1 phase causing prolonged cell cycle[68]. The discovery that $LXA_4$, but surprisingly not LXB4 despite its structural similarity to $LXA_4$ can bind and activate the AhR came by chance when a team headed by Bjeldanes LF wanted to explore and characterise the endogenous ligands responsible for binding to the AhR, allowing it to carry out its role in maintaining normal physiology within the cell. They did this using a varied collection of techniques including gel mobility shift analysis to show that $LXA_4$ can transform the AhR into a form capable of binding to the DNA, AhR competitive binding assays and northern blot analysis to prove that $LXA_4$ can cause a transient up-regulation of the mRNA in a AhR-responsive gene CYP1A1, involved in xenobiotic metabolism[69]. This indicates that $LXA_4$ is acting as a substrate for this cytochrome P450 isoform. The temporally-restricted expression of CYP1A1 suggests an autoregulatory loop for $LXA_4$ metabolism, a physiological feature in common with many AhR ligands. The finding that $LXA_4$ activates AhR not only for its own metabolism but also in disease states for immune regulation provides a fascinating insight in to how environmental pollutants may affect our immune systems.

## ALX Signalling Pathways

Classically, ALX activation leads to LTB4-induced accumulation of PSDP (polyisoprenoid presqualene diphosphate), inhibition of PLD (phospholipase D) and abrogation NADPH oxidase assembly thereby inhibiting superoxide anion generation by neutrohphils[70]. Jozsef and colleagues demonstrated that $LXA_4$, ATLs and their analogues inhibit pro-inflammatory genes such as IL-8[71] via an ALX-dependent peroxynitrite mediated signalling pathway. It is the reaction between nitric oxide and superoxide that leads to the formation of peroxynitrite, a hallmark of several pathologies and has been shown to regulate leukocyte intracellular signalling events that affect inflammatory responses[72,73]. It has also been shown that in human leukocytes stimulated with LPS, TNFalpha or IL-beta, peroxynitrite-induced IL-8 gene expression occurs via a NFkappaB and AP1 dependent pathway[74,75]. Knowing that IL-8 acts as a potent neutrophil chemoattractant[76] it can be concluded that the ability of LXs to modulate induction of IL-8 gene expression, via the inhibition of peroxynitrite formation, is a sturdy anti-inflammatory mechanism. ATL analogues also regulate an ALX dependent p38/MAPK cascade, known to promote chemotaxis, by inhibiting leukocyte-specific protein-1 phosphorylation and activation[77].

ALX plays a pivotal role in inflammation by down-regulating several pro-inflammatory pathways but also upregulates and/or promotes protective events key to the initiation of resolution. Serhan and colleagues have demonstrated the involvement of the transcriptional co-repressor NAB-1 in the resolution phase of inflammation. Three-way differential display PCR characterised ATLa responsive genes in neutrophils. The NAB1 gene was found to be positively and selectively regulated by ATLa and the NAB1 protein upregulated via a G-protein coupled receptor mediated pathway identified as ALX[78].

While most of the actions of LX and ATLs are mediated via an ALX-dependent mechanism, recent evidence from Aliberti and colleagues suggests an additional anti-inflammatory pathway involving a member of the suppressor of cytokine signalling SOCS-2 via the nuclear receptor aryl hydrocarbon receptor (AhR) in dendritic cells. They show that the LXs regulatory functions are mediated via the induction SOCS-2 expression and activity. *In vitro* treatment of mouse splenic DCs with $LXA_4$ and *in vivo* stimulation with Toxoplasma Gondii tachyzoite lysate, known to trigger endogenous $LXA_4$ generation, showed dependence of SOCS-2 expression on LXs and LX generating enzymes as well as the involvement of both ALX and AhR. LX induced SOCS-2 also regulates pro-inflammatory cytokine responses, leukocyte infiltration into the liver and brain and expansion of IFNgamma producing T cells. Evidence that LX induction of SOCS-2 is a general anti-inflammatory mechanism controlling several innate responses was shown in SOCS-2 deficient mice whose monocyte and neutrophil accumulation in the peritoneum infected with *T. gondii* was greatly enhanced. Finally, aspirin-induced anti-inflammatory effects showed a dependence on SOCS-2 in an *in vivo* model of peritonitis[79]. The intracellular signalling pathway of SOCS-2 induction remains unclear however. Could NAB1 induction be the missing upstream event leading to SOCS-2 transcription?

Several possible pathways through which aspirin exerts its powerful anti-inflammatory effects have been outlined with even more to enumerate and elucidate. As the concept of resolution has been alluded to, it is now appropriate to emphasise the importance of the active process of inflammatory resolution, without which acute inflammatory events would develop into chronic inflammatory conditions such as rheumatoid arthritis and asthma, to name but a few. The purpose of the immune system in fighting infection or other injury is to neutralise and eliminate injurious agents. Several naturally occurring mechanisms occur in self-resolving inflammatory states such as the inhibition of neutrophil recruitment by LXs, the increased chemotaxis of monocytes, increased phagocytosis of apoptosing neutrophils at a site of inflammation.

## RESOLVINS

Other pro-resolution factors or "braking signals" exist, and of most interest are the omega-3 fatty acid derived mediators called resolvins. These originate, not from arachidonic acid but from EPA (eicospentaneoic acid) and DHA (docosahesaenoic acid). Benefits of omega-3 derived poly-unsaturated fatty acid supplementation in certain human diseases have been suggested for over a quarter of a century. Schmidt and colleagues review beneficial effects such as potential anti-thrombotic, immunoregulatory and anti-inflammatory responses with regard to diseases such as arteriosclerosis, arthritis and asthma, as well as anti-tumour and anti-metastatic effect[80]. In 1999 results of a trial (GISSI-Prevenzione) evaluating the benefits of aspirin with and without omega-3 PUFA supplementation in patients recovering from myocardial infarctions revealed a significant decrease in mortality in the group taking the supplement[81]. The mechanisms by which fish oils exert their effects have been put forward and include inhibition of PG and LT synthesis, ability to act as a replacement substrate for the 5-LOX in order to produce less potent 5-series LTs or COX mediated conversion to anti-thrombotic prostanoids[80].

Keen to uncover the molecular bases for the protective actions of omega-3 PUFA, it was shown in a mouse model of self-resolving inflammation: a dorsal skin pouch model that allows for an accurate measurement of the extent of neutrophil infiltration and most importantly of lipid mediator generation during the resolution phase of inflammation[82,83]. From these studies a lipid mediator library was constructed and novel mediators, if present, were structurally analysed and organically synthesised. The novel mediators were classed as resolvins and docosatrienes (DT): 1) 18R resolvins from EPA (RevE1), 2) 17R series (aspirin triggered) resolvins from DHA (RvD1-RvD6), 3) 17S series resolvins from DHA (RvD1-RvD6), 4) DT from DHA and 5) their aspirin-triggered form 17 R series DT see reference ([84] for review and Figure 3). Indeed, exudates from murine dorsal air pouches generated novel lipid derived compounds in response to omega-3 and aspirin administration. These include 18R-HEPE and trihydroxy-containing compounds derived from EPA and, interestingly when COX-2 expressing human endothelial cells were treated with aspirin and pulsed with EPA a mixture of 18R-HEPE and 15-R HEPE was generated[85]. Similarly in mouse exudates given aspirin and DHA, a novel 17R D series of resolvins called 17R-HDHA, generated via the acetylation of COX-2[82], was also found to be generated by human microvascular endothelial cells. A final series of 17S resolvins was identified and found to be induced in the absence of aspirin from endogenous DHA. Refer to figure 3 for summary of resolvin and docosatriene pathways.

Regarding their anti-inflammatory properties, resolvins of the D series block TNFalpha induced IL-1beta transcripts and are potent regulators of neutrophil infiltration in the brain, skin and peritonitis *in vivo* as demonstrated by Hong and colleagues[86]. It was also demonstrated that both D and E classes of resolvins downregulated neutrophil infiltration, for review see reference[84]. It was also shown that the switch from S to R configuration induced by aspirin does not reduce the beneficial effects resolvins have on neutrophil infiltration[83]. In addition, it has recently been shown that RvE1 causes *in vivo* reduction in inflammation and protects against bone destruction in periodonitis[87]. These studies present EPA and DHA derived mediators, induced by aspirin or not, as potential targets in anti-inflammatory processes, particularly resolution. This novel anti-inflammatory pathway is triggered not only by aspirin but also diclofenac and indomethacin.

The existence of such compounds does not preclude the ability of LXs to participate in the resolution of inflammation. Indeed as described previously and hereafter, *in vitro* evidence demonstrates LXs' inhibitory effects on neutrophil chemotaxis and adhesion. Of biological relevance is complementary in vivo evidence demonstrating a central role for LXs in the control and resolution of periodontital diseases by controlling and preventing neutrophil-mediated injury, characteristic of inflammatory diseases such as periodontitis[88,89]. Indeed Serhan and colleagues have reviewed the importance of 15-LOX and LX related pathways in the protection against bone destruction in clinical settings such as osteoporosis and periodontitis[90].The fire that is LX and inflammation research is still blazing clearly suggests that LXs have applications in many different disease states, making them a therapeutic agent with a great deal of potential.

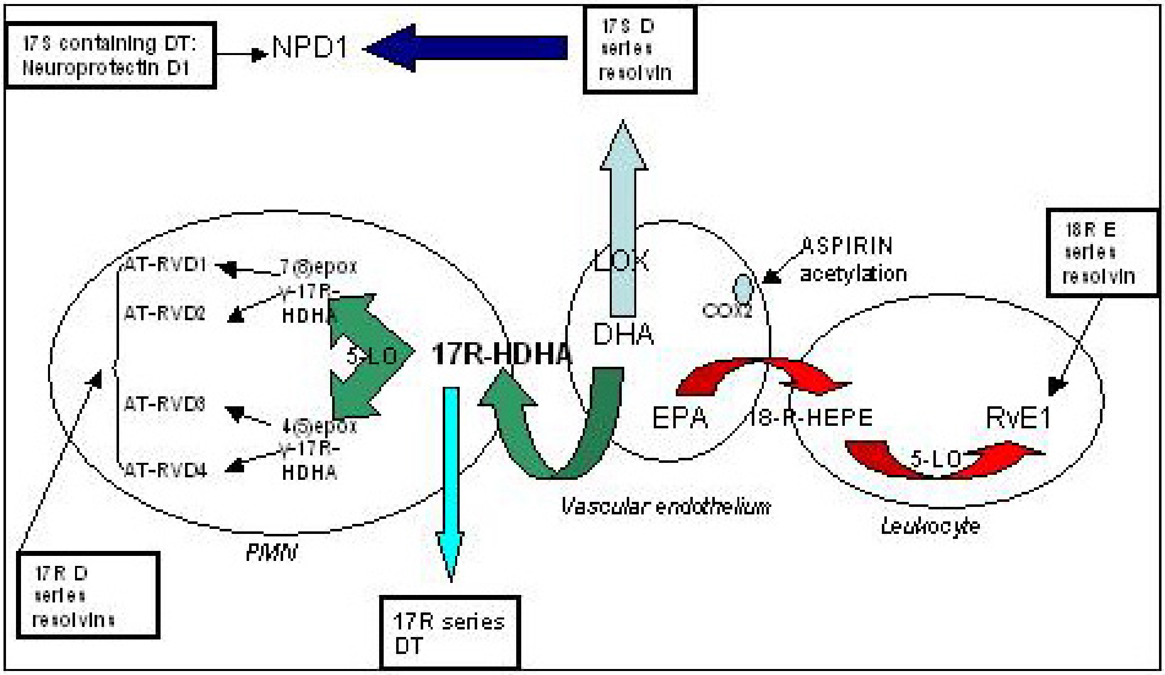

**FIGURE 3.** DHA is a source for 17R D series resolvins and EPA a source for 18R E series resolvin after COX-2 acetylation by aspirin. DHA also gives rise to 17R and 17S docosatrienes in the absence of aspirin. The latter can also induce neuroprotectin formation ie: NPD1.

## LXs and leukocyte-endothelial cell interaction

An aspect not yet alluded to and of much importance to aspirin induced anti-inflammation is the central role of nitric oxide and LXs in the control of the acute phase of inflammatory responses. Mechanisms by which LXs and epi-LXs participate in aspirin-induced anti-inflammatory effects are by dampening leukocyte-endothelium interactions[91], affecting leukocyte diapedesis[42] and chemotaxis[92], to mention but a few. $LXA_4$ stable analogues modulate L-selectin and CD11/CD18 on resting and stimulated leukocytes as well as inhibit neutrophil adhesion to human coronary artery endothelial cells by reducing CD11/CD18 expression[93]. Knowing that both nitric oxide and LXs are anti-inflammatory in their ability to regulate leukocyte recruitment to inflammatory sites, it has been hypothesised that aspirin may exert its anti-inflammatory effects via ATL-mediated induction of nitric oxide[94,61,95]. A reduction in 15-epi-$LXA_4$ synthesis, be it due to selective COX-2 inhibition or ALX antagonism, enhanced neutrophil-endothelial cell interaction *in vitro*, and this correlates with the observations that nitric oxide down-regulates leukocyte/endothelial interaction via inhibition P-selectin[96] and CD11/CD18 expression[97] and the fact that anti-adhesive effects of a nitric oxide donor were unchanged even though 15-epi-$LXA_4$ synthesis was inhibited, suggests an ATL involvement in the generation of anti-inflammatory nitric oxide[98,99].

Along the lines that nitric oxide could be the main culprit carrying out aspirin induced anti-inflammatory effects, published data from Gilroy and colleagues showed elevated plasma nitrite/nitrate and reduction in local inflammation in rats bearing acute pleuritis after treatment with a single anti-inflammatory dose of aspirin. Treatment with traditional NSAIDs indomethacin, salycilate and piroxicam confirmed that these effects were aspirin specific. Such observations were particularly important in view of nitric oxide's well-established role in prevention of leukocyte/endothelial cell interaction during acute inflammation. A complementary series of experiments showing that inhibition of aspirin-induced nitric oxide blocked its ability to down-regulate cell interaction emphasised the dependence of the induction of aspirin's anti-inflammatory effects on aspirin-induced nitric oxide. In a model of mouse IL-1beta induced peritonitis the anti-inflammatory effect of aspirin was abrogated in mice deficient of either the constitutive (eNOS) or inducible (iNOS) forms of the nitric oxide synthase enzymes. In both settings reduced plasma nitric oxide correlated with increased numbers of cells accumulating in the peritoneal cavity. They later demonstrated that the nitric oxide generated in response to aspirin treatment was via the aspirin triggered lipid intermediate 15-epi-$LXA_4$. Indeed, using intravital microscopy on IL-1beta stimulated mouse mesentry, it was shown that aspirin and 15-epi-$LXA_4$ inhibit leukocyte trafficking in a nitric oxide-dependent manner. Not only did aspirin inhibit leukocyte-endothelial interaction similarly to the way nitric oxide did in wild-type mice, but the ability of aspirin and 15-epi-$LXA_4$ to down-regulate leukocyte-endothelial interaction in mice deficient in both eNOS and iNOS was reduced compared to wild type controls[56]. A convincing conclusion was drawn: nitric oxide mediates the anti-inflammatory effects of aspirin, via the acetylation of COX-2 in endothelial cells or circulating leukocytes, with subsequent biosynthesis of 15-epi-$LXA_4$ which in turn elicits nitric oxide synthesis from both eNOS and iNOS.

These studies have confirmed a role for epi-LXs in the generation of nitric oxide, which is known to play a pivotal role in the early recruitment stages of inflammation. They also underline the fact that both eNOS and iNOS are targeted by ATLs and are both required for the control of leukocyte trafficking in a possibly sequential fashion (ie. eNOS setting the stage of subsequent iNOS induction). The mechanism involved in the regulation of these enzymes by aspirin and epi-LXs remains enigmatic. The possibility of some kind of cross talk between eNOS and iNOS in this setting is of interest. Indeed, there is evidence that NFkB in murine macrophages is affected by low levels (from eNOS) or high levels (from iNOS) of nitric oxide, enabling up- or down- regulation of NFkB respectively. Connelly and colleagues recently showed that LPS-stimulated bone marrow derived macrophages from eNOS knockout mice present greatly reduced NF-κB activity and iNOS expression compared to wild type cells[100]. This observation was followed by the demonstration that soluble guanylate cyclase (sGC), known to be induced by nitric oxide levels approximating those from eNOS, is responsible for increased iNOS induction. This suggests an indirect induction of iNOS by eNOS via a sGC-dependent NFkB activation pathway. A critical role for this NO/cGMP pathway in cytokine signaling of iNOS expression was also shown in human mesangial cells[101]. If this theory is to be accepted, it wasn't surprising that the selective inhibition of eNOS derived nitric oxide synthesis by an inhibitor such as L-NAME would result in subsequent down-regulation of iNOS induction. Furthermore, the effect was reversed using a nitric oxide donor. Such *in vitro* findings could explain why eNOS $^{-/-}$ and iNOS$^{-/-}$ mouse nitric oxide plasma levels are lower than in aspirin-treated wild type animals. Thus, eNOS activation may be a primordial event contributing to the anti-inflammatory actions of aspirin and LXs.

We have also showed that naïve mice responded to aspirin and 15-epi-$LXA_4$ treatment with increased plasma nitric oxide levels, suggesting that the machinery responsible for inducing aspirin triggered nitric oxide, identified as the COX-2/5 LOX pathway is constitutively expressed, even though classically COX-2 was not thought of as a constitutive enzyme[56]. It has been put forward, considering the large size of the vasculature, that "hot spots" which, under stress, can generate COX-2 derived epi-LXs after aspirin treatment[85]. This theory is strengthened by *in vitro* models of shear stress mimicking the wall shear encountered in the vasculature, showing sustained COX-2 expression[102]. Although there is also evidence of COX-2 expression in leukocytes, the exact source of the constitutive enzyme has to be established[103]. The involvement of nitric oxide in the mediation of aspirin induced anti-inflammatory effects is an extremely hot topic currently under investigation.

## CONCLUDING REMARKS

This review reveals many, but certainly not all, of the elusive properties of aspirin. Its ability to affect diseases of diverse aetiologies suggests that aspirin most likely will have many modes of action depending on the disease state. Vane's groundbreaking work exposing aspirin as an inhibitor of PG synthesis was one of the first branches that rekindled the fire that lay gently smoldering. Indeed, alternative and/or additional mechanisms have been investigated and dismissed over the past three decades. Such mechanisms include the inhibition of pro-inflammatory signaling pathways, gene expression and other factors distinct from eicosanoid biosynthesis, in addition to the triggering of endogenous anti-inflammatory/pro-resolution signals including the LX family of eicosanoids. The aspect of pro-resolution of inflammation is key to the understanding that perhaps anti-inflammation is not simply due to the inhibition of pro-inflammatory mediators but most likely to complementary induction of anti-inflammatory pathways. Exploitation of the anti-inflammatory properties of compounds such as the LXs and resolvins, whose syntheses can be triggered by aspirin, is primordial in that the optimization and understanding of their mechanisms of action will enable the development of clinically novel and extremely efficacious prophylactic and/or therapeutic anti-inflammatory drugs based on the multiple and variable anti-inflammatory properties of aspirin during both the onset and resolution phases of acute or chronic inflammatory responses. The investigation of aspirin's unique behavior with regards to inflammation is continuing.
